# Supplementary material for: An open-source framework for large-scale, flexible evaluation of biomedical text mining systems
Source: J Biomed Discov Collab. 2008 Jan 29;3:1. doi: 10.1186/1747-5333-3-1 (PMC2276192; doi:10.1186/1747-5333-3-1)
Supplement: Additional file 1 — Evaluation platform source code. The additional file contains the software evaluation framework discussed in this paper. The most current release can be downloaded from SourceForge.net [1]. See the accompanying README file for instructions on installation and use. Also included in the distribution is a collection of UIMA wrappers for some commonly used BioNLP tools and annotated corpora. [file 1747-5333-3-1-S1.zip › bionlp_uima_repository-1.0.1/docs/api/src-html/edu/uchsc/ccp/util/nlp/ei/EIFileBasedSystem.html]

```
001    /*
002     * Created on Aug 15, 2005
003     *
004     * TODO To change the template for this generated file go to
005     * Window - Preferences - Java - Code Style - Code Templates
006     */
007    package edu.uchsc.ccp.util.nlp.ei;
008    
009    import java.util.List;
010    
011    import edu.uchsc.ccp.util.nlp.annotation.TextAnnotation;
012    
013    /**
014     * @author williamb
015     *
016     */
017    public interface EIFileBasedSystem extends EISystem{
018            
019            /**
020             * 
021             * @param inputFilename - the name of the tagged file which needs to be parsed to find the gene hits
022             * @return - ArrayList of TextAnnotations
023             */
024            //public abstract ArrayList returnGeneHits(String taggedTextFilename, String originalTextFilename, ArrayList documentSectionIDs, ArrayList documentSectionLengths);
025        public abstract List<TextAnnotation> getEntitiesFromFile(String originalTextFilename);
026            
027        
028            /**
029             * 
030             * @return - String containing the name of the file where the system's output is stored
031             */
032            public abstract String getOutputFilename();
033            
034            
035    //      /**
036    //       * 
037    //       * @param inputFile - name of the file containing text to be tagged
038    //       * @throws Exception
039    //       */
040    //      public abstract void acceptFileInput(String inputFile) throws Exception;
041    //      
042    //      
043    //      /**
044    //       * 
045    //       * @param s - string containing the text to be tagged
046    //       * @throws Exception
047    //       */
048    //      public abstract void acceptStringInput(String s, String documentID) throws Exception;
049            
050            
051    }
```
